# Quantification of Cardiovascular Disease Biomarkers in Human Platelets by Targeted Mass Spectrometry

**DOI:** 10.3390/proteomes5040031

**Published:** 2017-11-15

**Authors:** Sebastian Malchow, Christina Loosse, Albert Sickmann, Christin Lorenz

**Affiliations:** Leibniz-Institut für Analytische Wissenschaften-ISAS-e.V., 44139 Dortmund, Germany; sebastian.malchow@isas.de (S.M.); christina.loosse@isas.de (C.L.); albert.sickmann@isas.de (A.S.)

**Keywords:** quantitative proteomics, mass spectrometry, parallel reaction monitoring, cardiovascular diseases, platelets

## Abstract

Platelets are known to be key players in thrombosis and hemostasis, contributing to the genesis and progression of cardiovascular diseases. Due to their pivotal role in human physiology and pathology, platelet function is regulated tightly by numerous factors which have either stimulatory or inhibitory effects. A variety of factors, e.g., collagen, fibrinogen, ADP, vWF, thrombin, and thromboxane promote platelet adhesion and aggregation by utilizing multiple intracellular signal cascades. To quantify platelet proteins for this work, a targeted proteomics workflow was applied. In detail, platelets are isolated and lyzed, followed by a tryptic protein digest. Subsequently, a mix of stable isotope-labeled peptides of interesting biomarker proteins in concentrations ranging from 0.1 to 100 fmol is added to 3 μg digest. These peptides are used as an internal calibration curve to accurately quantify endogenous peptides and corresponding proteins in a pooled platelet reference sample by nanoLC-MS/MS with parallel reaction monitoring. In order to assure a valid quantification, limit of detection (LOD) and limit of quantification (LOQ), as well as linear range, were determined. This quantification of platelet activation and proteins by targeted mass spectrometry may enable novel diagnostic strategies in the detection and prevention of cardiovascular diseases.

## 1. Introduction

### 1.1. Platelets Are Key Players in the Generation and Progression of Cardiovascular Diseases

Cardiovascular diseases (CVDs) encompass disorders of the heart and blood vessels. They are the leading cause of death worldwide and account for 17.5 million deaths per year, an estimated 31% of all deaths worldwide [1], and are therefore a high burden to public health and health systems. In recent years, platelets have emerged as important markers for various types of cardiovascular diseases. These anucleated blood cells are involved in thrombosis and hemostasis [2]. While circulating in the blood stream, they have a lifespan of 7–10 days with only minor interaction with other blood components. Due to their pivotal role in physiology and pathology, platelet functions are regulated tightly by numerous factors, which have either stimulatory or inhibitory effects. A variety of agonists including collagen, fibrinogen, adenosine diphosphate (ADP), von Willebrand factor (vWF), thrombin, and thromboxane promote platelet activation, adhesion, and aggregation by utilizing multiple intracellular signal transduction pathways. Whereas collagen seems to be the main effector of activation, ADP enhances the responses of different agonists [3]. Upon vessel lesions, vWF and collagen exposition, as well as thrombin generation, platelets are captured at the endothelial cells and activated through the respective surface receptors. The pathways are highly interconnected, consequently leading to platelet shape change from discoid to dendritic, integrin activation, and granule release. Besides molecular stimuli, external forces such as shear stress promote activation processes. The activation propagation occurs when additional platelets are recruited to the growing hemostatic plug by released or secreted secondary agonists—thromboxane or ADP, for example. Finally, the thrombus formation is initiated by the onset of the coagulation cascade. In circulating platelets, continuous exposure of inhibitory factors such as nitric oxide and prostacyclin ensure the resting, non-activated state. A well-balanced equilibrium between those opposing processes is assumed to be essential for normal platelet and vascular function. Consequently, a shifted equilibrium promotes uncontrolled platelet aggregation, and inflammatory and/or bleeding disorders.

### 1.2. Targeted Mass Spectrometry as a Clinical Tool-Kit for Future Diagnostics

To reveal the molecular mechanism of cardiovascular diseases in detail and to develop new diagnostic strategies, a thorough identification and characterization of potential biomarkers in human blood is required. Today, diagnosis is feasible usually in an acute state of the disease, e.g., after a myocardial infarction, when blood tests for, e.g., elevated troponin can be performed. Most CVDs can be prevented by addressing behavioral risk factors. However, people with CVDs or who are at high risk need early detection and management. Typical markers for the risk evaluation are, e.g., cholesterol and lipids (i.e., LDL, HDL) [4,5]. Furthermore, elevated protein marker levels of, for example, the C-reactive protein [6], apolipoprotein A1 and B [7], as well as troponin and fibrinogen [8] are indicative. To date, protein markers are diagnosed routinely by the Enzyme-linked Immunosorbent Assay (ELISA). This analytical method depends highly on antibody specificity and faces several other issues such as cross-reactivity and undetected isoforms while only being able to obtain very limited protein data.

Over the last years, mass spectrometry has gained significant attention for relative and absolute quantification of proteins in different matrices, as well as post-translational modifications (PTMs). Due to limited protein synthesis in platelets, PTMs play a pivotal role in signal transduction during platelet activation and inhibition. Notably, through advances in sample preparation, mass spectrometry instrumentation, and data analysis software, the multiplexed absolute quantification of proteins via proteotypic peptides in large sample cohorts became feasible. Moreover, mass spectrometry has emerged as the method of choice when it comes to identifying platelet proteins. Previously, we were able to identify and quantify around 4000 platelet proteins, among them novel receptors and signaling proteins [9]. Furthermore, more than 4000 phosphorylation sites were identified [10,11] and further studies were performed in order to continue elucidating platelet PTMs, such as glycosylation [12,13,14] and palmitoylation [15,16,17]. Therefore, mass spectrometry is a promising tool for establishing assays for CVD diagnosis as it allows the quantification of several proteins in parallel.

**Figure 1:**
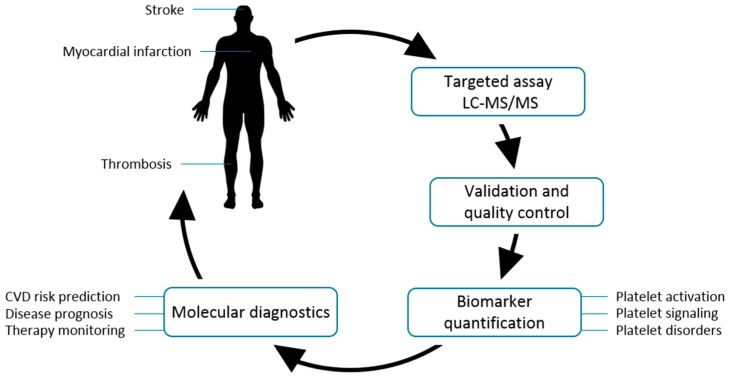
Quantification of potential cardiovascular disease biomarkers by targeted proteomics. In order to identify new biomarkers for cardiovascular disease (CVD), platelet proteins are used for targeted nanoLC-MS/MS analysis. For reliable results, these measurements are performed under standardized conditions, including method validation and quality controls. Quantification is performed for targets related to platelet activation, platelet signaling, and platelets disorders. Determined proteins might be used for molecular diagnostics, allowing CVD risk prediction, disease prognosis, and therapy monitoring, helping to prevent common CVDs such as stroke, myocardial infarction, and thrombosis.

In this study, proteins related to platelet activation and functional disorders were quantified by parallel reaction monitoring (PRM) mass spectrometry and a targeted assay in combination with stable isotope-labeled (SIL) standard peptides (Figure 1). Looking ahead, this approach may represent novel diagnostic strategies in the prevention of cardiovascular diseases.

## 2. Materials and Methods 

### 2.1. Platelet Isolation

Platelets were isolated as described in Beck et al., 2014 [11] with slight modifications: Instead of fresh blood preparations, platelet concentrates from five healthy donors were used. After the separation of plasma and platelets via centrifugation, platelets were washed and concentrated in washing buffer (10 mM sodium citrate, 0.7 mM citric acid, 14 mM glucose, 1.5 mM EDTA, 145 mM sodium chloride, 5 mM potassium chloride, 1 mM magnesium chloride).

### 2.2. Sample Preparation

Samples were lyzed in 30 μL lysis buffer (150 mM NaCl, 50 mM Tris-HCl, 4% (*w*/*v*) SDS, PhosStop, cOmplete Protease inhibitor) and protein concentration was determined using bicinchoninic acid (BCA) assay. Subsequently, 150 μg sample was reduced with 10 mM DTT for 30 min at 60 °C and alkylated with 20 mM iodacetamide in the dark for 30 min at room temperature. Proteins were precipitated with ice-cold ethanol for 1 h at −40 °C. After centrifugation (21,000× *g*, 30 min, 4 °C) the pellet was resuspended in ice-cold acetone and sedimented again (21,000× *g*, 15 min, 4 °C). The dried sediment was resolubilized in 30 μL 6 M GuHCl. The sample was diluted 1:30 with digestion buffer (final concentration: 5% acetonitrile (ACN), 2 mM CaCl_2_, trypsin:protein 1:20, in 50 mM NH_4_HCO_3_) and incubated overnight at 37 °C. Digestion was stopped by adding TFA to a final concentration of 1%. Afterwards, samples were desalted by C18 solid phase extraction. After desalting, SIL peptides were spiked in the samples in a concentration range from 0.1 to 100 fmol.

### 2.3. Synthesis of SIL Peptides

SIL peptides were synthesized in-house using the Fmoc solid-phase peptide synthesis (SPPS) strategy on a Syro II synthesis unit (MultiSynTech, Witten, Germany). Peptides were cleaved from the solid support, precipitated, rinsed, and purified. After drying and solubilization, peptides were purified by mass-selective fractionation using reversed-phase chromatography. The purified peptides were lyophilized and resolubilized for quantification by amino acid analysis (AAA). AAA consisted of gas-phase acid hydrolysis and fluorescence labeling, and was performed in-house using the Acquity UPLC H class system (Waters, Milford, MA, USA). Purified and quantified peptides were stored in 30% ACN and 0.1% TFA at −40 °C.

### 2.4. Mass Spectrometry Analysis

Platelet digests of 3 μg in 15 μL 0.1% TFA were separated with an Ultimate 3000 RSLCnano Pro Flow system (Thermo Scientific, Waltham, MA, USA), concentrated on an Acclaim PepMapTM 100 C18 trapping column (2 cm length, 100 μm i.d., 5 μm particle size, pore size 100 Å, Thermo Scientific), and separated on an Acclaim PepMap^®^ RSLC C18 column (50 cm length, 75 μm i.d., 2 μm particle size, pore size 100 Å, Thermo Scientific) heated to 60 °C. Sample preconcentration was performed for 5 min with a flow of 20 µL/Min and 0.1% TFA. A 120 min gradient from 3% to 45% solvent B (84% ACN/0.1% FA, 250 nL/Min) was applied for peptide separation. The nanoHPLC was coupled to a Q Exactive Hybrid Quadrupole-Orbitrap Plus mass spectrometer (Thermo Scientific) and eluting peptides were ionized directly via a silica emitter (FS360-20-10-D-20, New Objective, Ringoes, NJ, USA) of the nanoESI source. Peptides of interest were analyzed using a scheduled parallel reaction monitoring method, which was performed with a resolution of 17,500, a maximum injection time of 50 ms, and an automatic gain control (AGC) value of 3 × 10^6^. The isolation window was 0.4 *m*/*z* for the respective masses of endogenous and SIL peptides.

### 2.5. Data Evaluation Using Skyline and R

For the analysis of the raw data, we used Skyline 64 bit 3.6.0.10570 [18]. The data was then exported to *.csv and imported into R [19]. Here, we used R Studio 0.99.903 and R 3.3.1 “Bug in Your Hair”. The linear regressions and concentration determinations were performed with the functions *lm* and *predict* of the R *stats* package which is part of R. The various functions of the *plyr* and *ggplot2* packages were used for data handling and plotting, respectively [20,21].

## 3. Results and Discussion

### 3.1. Establishment of the Targeted Mass Spectrometry-Based Assay

Besides pathological platelet counts in thrombocytopenia or thrombocytosis, dysfunction of platelets, due to either intrinsic defects or extrinsic factors, are accountable for cardiovascular diseases. The underlying mechanisms and molecules that are affected include (i) platelet function defects due to abnormalities in platelet receptors; (ii) platelet granules; (iii) membrane phospholipids; and (iv) signal transduction cascades. Those functional disorders provoke either an increased bleeding rate or thrombotic conditions, e.g., coronary heart disease, cerebrovascular disease, deep vein thrombosis, stroke, and pulmonary embolism [1].

The proteins potentially involved in the progression of thrombotic conditions were chosen based on our previous studies [9,10,11,22] combined with knowledge from public data sets. The target proteins for quantification are related to platelet activation and signaling, e.g., PKC [23] and LCP2 [24]; platelet secretion, e.g., STX4 [25] and STIM1 [26]; and platelet aggregation, e.g., ITA2 [27]. In order to obtain reliable and comparable results, high demands are placed on study design, sample preparation, assay development, and data analysis when conducting clinical proteomic studies in general and in particular when using platelets as the study object [28,29].

Among various quantification methods developed in the field of proteomics in recent years, multiple and parallel reaction monitoring (MRM and PRM) methods combined with the use of isotope-labeled standards are seen as the most reliable in terms of accuracy [30,31,32,33]. To guarantee the quality and validity of the results, the application of such complex quantitative methods must be executed carefully. Thus, the development of targeted mass spectrometry assays for clinical applications is a challenge.

**Figure 2:**
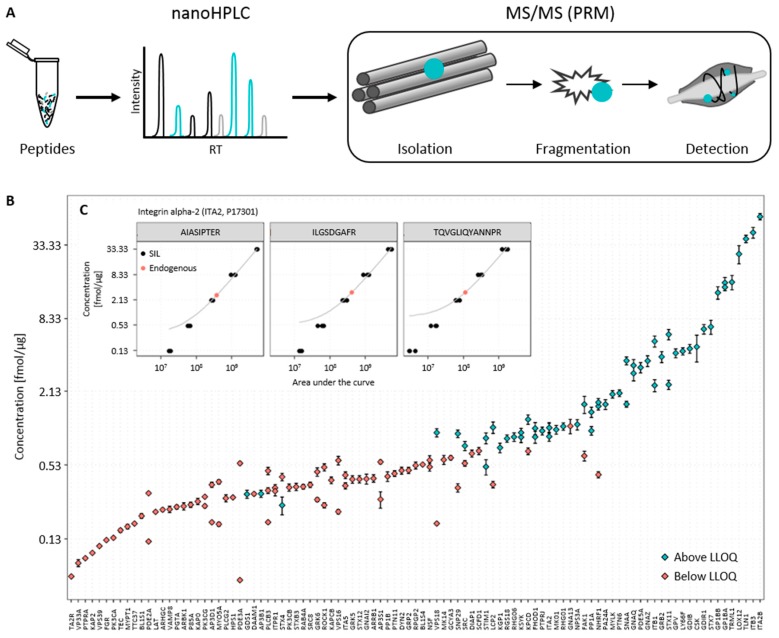
Quantification of platelet proteins by an internal standard curve. (**A**) Proteomics workflow. The prepared peptide samples were separated by nanoHPLC and measured subsequently with nanoESI-MS parallel reaction monitoring (PRM). Peptides of interest were isolated sequentially in a quadrupole, then fragmented, and finally peptide fragments detected in an orbitrap mass analyzer; (**B**) Distribution of endogenous concentration of quantified proteins. In total, 133 peptides corresponding to 99 proteins in human platelets were quantified; (**C**) Quantification of Integrin alpha-2 (ITA2) via an internal calibration curve and least squares linear regression. The dots represent the measured areas of the quantifier transition of the stable isotope-labeled (black) and endogenous peptides (red) plotted against the known or determined peptide concentration. Plotted in grey is the straight line fitted to the measured area under the curve of the quantifier transition of stable isotope-labeled peptide using least squares linear regression.

Here, peptides and proteins were identified via discovery mass spectrometry methods. From these identified peptides, a specific subset matching the proteins of interest were chosen. Afterwards, peptide samples were separated by nanoHPLC and measured subsequently using PRM mass spectrometry (Figure 2A). To our knowledge, those peptides are proteotypic and do not contain any sites or sequences which are prone to chemical modification during sample processing [34,35]. Furthermore, the selection was based on empiric parameters such as highest mass spectrometric response and interference from other peptides or sample matrix [36]. We implemented the general standards as follows: From the data dependent mass spectrometry (ddMSMS) measurements, up to ten of the most intense peptides per protein were selected. All selected peptides were unique on the proteome level and did not contain missed cleavages, ragged ends, methionine, or a series of asparagine and glycine, or carry glutamine or glutamic acid on the N-terminus. Subsequently, peptides were measured using PRM mass spectrometry. The peptide identity was checked by comparing fragmentation patterns to the discovery spectra. During this process, the quality and validity of the identification was taken into consideration. Up to three peptides were chosen based on the mass spectrometry response, validity of identification, and low interference of other peptides or matrix, followed by a synthesis of corresponding SIL peptides. Starting with a set of 214 platelet proteins of interest, the described process led to a panel of 375 purified and quantified SIL peptides corresponding to 151 proteins, which built the scaffold for developing targeted quantitative mass spectrometry assays.

### 3.2. Determination of Endogenous Concentration of Relevant Platelet Proteins

Of the large panel of peptides and proteins mentioned before, we quantified a subset of 99 proteins and 133 peptides via an internal calibration curve in one platelet reference sample. Using calibration curves is a common procedure for an absolute quantification, although the curve preparation can vary [37,38,39]. As a reference sample we used a mix of platelets derived and pooled from five healthy donors. The calibration curve was established using a mixture of SIL peptides with a concentration of 20 fmol/μL each. Subsequently, a dilution series was applied to reach final concentrations of 100, 25, 6.25, 1.563, 0.391, and 0.098 fmol in 3 μg platelet matrix. After setting up a scheduled PRM method, the concentration curve was measured three times from lowest to highest concentration including a platelet sample without spike-in SIL peptides.

The raw measurements were imported into Skyline [18] and all measurements reviewed and inspected manually. First, the top 3–5 transitions exhibiting the highest response and lowest interference were chosen. Interferences manifested in high mass deviation, or unusual peak shape. Second, the detection limit of the selected peptide was determined. This detection limit was defined as the concentration in which at least three transitions of the peptide were measureable with a similar area upon all replicates. The transition showing the highest response was considered as the quantifier transition, the others as qualifiers [40]. Any peptide not matching this definition was discarded. Finally, the integration of each peptide in all measurements was inspected manually and exported from Skyline. Further data analysis was done in R. The export listed the area under the curve for each transition of each peptide in each replicate, which resulted in a dataset of more than 26,000 single data points. This dataset was reduced by selecting the quantifier transition for each peptide and discarding all qualifier transitions. The quantifier transitions of the SIL peptides were determined in the first replicate of the 100 fmol measurement. This knowledge was then applied to all other replicates and endogenous peptides, which reduced the dataset by a factor of five. To calculate the concentrations of the endogenous peptides, we determined the linear relationships between the concentrations and the areas of the quantifier transition for each isotope-labeled peptide by least squares regression (see supplemental Table S1). An example is shown in Figure 2C. In order to determine the concentration, the linear relationship was applied to the measured areas of the endogenous quantifier transition. Because the areas of the endogenous peptides were obtained in 21 measurements, all resulting concentrations were combined and displayed as a mean ± standard deviation. The results of the quantification of selected platelet proteins are shown in Figure 2B and listed in the supplemental Table S2.

### 3.3. Data Analysis and Quality Control

In order to calculate the linear equation fitting the measured data, the areas of the SIL peptides were used for least squares regression. To calculate the endogenous concentrations, the areas of endogenous peptides of all replicates and the linear regression were used. From single concentrations, the mean concentration, corresponding standard deviations, and cofactors of variance were calculated. Overall, we determined the endogenous concentration of 133 peptides from 99 proteins related to platelet dysfunction or activation processes in a reference sample. Following the initial concentration determination, we assessed the accuracy and precision of the original data and the quantification. The precision was mainly assessed in the original data. Therefore, we determined the mean area and coefficients of variation (CV) of each peptide in each concentration. The usual CV% was ~10%; measurements with a value higher than 15% were inspected manually in Skyline and either removed or corrected accordingly. Despite the rigorous data review described above, most of the outliers could be explained by inaccurate peak integration and were corrected. Because the quantification accuracy mainly depends on the linear relationship between the area under the curve and the concentration, we investigated how well the linear relationships explain the original data. Thus, we used the residuals, which described the differences between the given concentration and the concentration predicted by linear regression by the vertical distance between the regression curve and the concentration of the SIL peptide in Figure 2C. The residual difference, shown as a percentage, was used to determine the lower limit of quantification (LLOQ). The smallest concentration with a mean residual percentage lower than 25% was determined as the LLOQ (see supplemental Table S3). Applying the determined LLOQs to the quantification of our targets showed that many peptide concentrations were below the LLOQ. However, we were able to determine the absolute concentration of 57 peptides and 45 corresponding platelet proteins with high accuracy and precision (Table 1). The quantifiable concentrations of target peptides spanned a range of 0.5 to 126 fmol/μg.

### 3.4. Increased Multiplexing Capability by Optimized Liquid Chromatography

The parallel quantification of more than one hundred peptides and their corresponding proteins is a great benefit compared with other methods like the limited Enzyme-linked Immunosorbent Assays (ELISA) [41]. Furthermore, it is valuable for the reduction of instrument time and ultimately the costs of an assay to process as many peptides as possible in a single, reliable assay. Besides practical concerns in assay development and data analysis, instrument factors limit multiplexing. To guarantee a precise determination of a peak area, and therefore the quantification in general, a minimum of ten measuring points over a peak is seen as a general rule of thumb. Due to a dwell time of ~50 ms per analyte and a peak width at half maximum (FWHM) of ~9 s, the multiplexed acquisition is limited to ~18 precursors and ~9 SIL and endogenous precursor pairs. Taking into account that the peak is around two times broader than the FWHM, a maximum of 35 precursors is reasonable. However, the mentioned numbers are strongly dependent on the used methods and instrumentation. The careful scheduling of acquisition times of specific precursors to specific times overcomes this limitation and achieves the numbers mentioned above, allowing a simultaneous analysis of hundreds of peptides [42,43]. Also, the amount of peptides eluting from the column at a certain time is not distributed equally. The optimization of the gradient leads to a changed analyte distribution, which was used by Moruz et al. [44,45] to improve the number of identifications in ddMSMS measurements. We applied this technique to improve the multiplexing of PRM assays.

**Figure 3:**
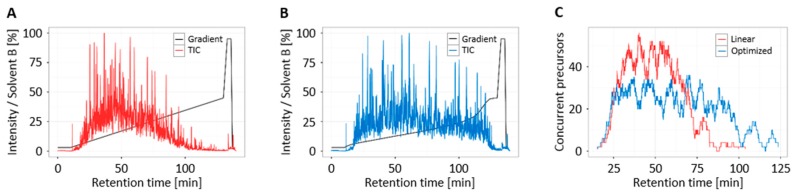
Gradient optimization for an increased multiplexing. (**A**) Total ion chromatogram (TIC) of a platelet digest using a linear gradient; (**B**) TIC of the platelet digest using the optimized gradient; (**C**) Comparison of the combined scheduling of 266 peptide pairs.

The original chromatographic gradient was linear from 3% B to 45% B (84% ACN, 0.1% FA) in 120 min. The MS1 spectra of a ddMSMS measurement and the software gradient optimizer, developed by the Käll lab, was used to find an optimized gradient. The new gradient is very shallow with two steep sections in the beginning and the end (for details see supplemental Table S4), and gave a more even distribution of eluting peptides. A comparison of the resulting total ion chromatograms is shown in Figure 3.

Again, we chose SIL subsets for the targeted experiments. These two subsets contained 130 and 136 peptides each. Digested platelets were spiked with a peptide mixture containing all peptides, resulting in samples with 20 fmol of each SIL peptide in a 2 μg platelet digest. Both peptide sets were measured in triplicate using the normal and optimized gradient. The combined scheduling of the two peptide sets is shown in Figure 3C. Applying 4-minute retention time windows for the retention time scheduling resulted in a maximum of 55 concurrent precursors when using the original linear gradient. The optimized gradient led to a reduction of approximately 20 precursors to a maximum of 35 concurrent precursors and a nearly two-fold increase of data points across the peak. Thus, the optimization allowed a two-fold increase of the multiplexing and reduced the instrument time in each run significantly.

## 4. Conclusions

Today, only a few protein biomarkers are used clinically in routine diagnostic strategies to detect cardiovascular diseases. When casting valid clinical biomarkers, the appropriate study design, sample type, and analytical method is of high relevance [46]. In collaboration with clinicians, well-characterized samples (i.e., of known gender, age, diseases, treatments/daily medications, and lifestyle) as well as appropriate controls need to be selected. Furthermore, the number of tested samples needs to be defined properly to gain statistical relevance and to detect changes confidently. The mass spectrometry-based quantification of proteins needs to be an important methodology to elucidate quantitative changes in health and disease. Hundreds of proteins can be quantified by using a targeted mass spectrometry approach. In order to gain reproducible and reliable results, similar high requirements are demanded of the technical part, e.g., used methods and workflows need to be standardized. Wegler et al. compared obtained results for the same sample from 6 different laboratories and revealed differences in determined protein concentrations [47]. Setting up a standardized method includes method validation to ensure accuracy, precision, sensitivity, specificity, and linearity [48]. Defining and characterizing these parameters is the key to constant quality and validity, which is crucial to a routine laboratory. Due to the increasing amount of information on platelet function in thrombotic diseases and platelet disorders, precise diagnoses and therapeutic monitoring are feasible. It can be assumed that the availability of the here-developed assay for platelet proteins will lead to significant advances in enlightening the molecular processes during the generation and progression of thrombotic events. In the future, we aim to perform those measurements under accredited conditions, i.e., we will implement a quality management system in our laboratory and used methods will be validated and performed with SOPs (standard operating procedures). Furthermore, measurements will be extended to several thousand well-characterized clinical samples. In collaboration with clinicians, the obtained values will be used to correlate protein amounts to, e.g., disease or disease state and lead to the revelation of altered proteins and/or protein patterns specific for one disease. To conclude, the here-presented workflow for targeted proteomics can direct the establishment of novel and feasible clinical assays to come.

## Figures and Tables

**Table 1:** Endogenous concentration of selected platelet peptides. All quantifiable peptides (concentration ≥ LLOQ (lower limit of quantification)) with their respective concentration in fmol/μg, standard deviation, coefficient of variation (CV), and LLOQ are shown. The concentration was calculated as the mean of all measurements. Proteins are grouped according to their functional classification.^1^ Uniprot accession for each protein; ^2^ Protein name; ^3^ Protein name abbreviation; ^4^ Analyzed peptide sequence; ^5^ Calculated concentration of endogenous peptide; ^6^ Standard deviation of calculated concentration; ^7^ Coefficient of variation of calculated concentration; ^8^ Lower limit of quantification of each peptide.

Protein Accession ^1^	Name ^2^	Short Name ^3^	Peptide Sequence ^4^	Concentration (fmol/μg) ^5^	Standard Deviation ^6^	CV (%) ^7^	LLOQ (fmol/μg) ^8^
**Platelet activation and signaling**							
P41240	Tyrosine-protein kinase CSK	CSK	HSNLVQLLGVIVEEK	14.7	3.6	24.7	2.1
Q05397	Focal adhesion kinase 1	FAK1	TLLATVDETIPLLPASTHR	5.0	0.8	16.0	2.1
Q9Y613	FH1/FH2 domain-containing protein 1	FHOD1	GDGEPVSVVTVR	2.7	0.3	11.1	0.5
Q9Y613	FH1/FH2 domain-containing protein 1	FHOD1	HLGTAGTDVDLR	3.2	0.4	11.3	2.1
P50395	Rab GDP dissociation inhibitor beta	GDIB	DLGTESQIFISR	14.2	1.2	8.2	2.1
P52565	Rho GDP-dissociation inhibitor 1	GDIR1	AEEYEFLTPVEEAPK	20.5	1.8	8.9	2.1
P52306	Rap1 GTPase-GDP dissociation stimulator 1	GDS1	SVAQQASLTEQR	0.9	0.1	7.6	0.5
P50148	Guanine nucleotide-binding protein G(q) subunit alpha	GNAQ	VSAFENPYVDAIK	8.9	1.2	13.3	2.1
P50148	Guanine nucleotide-binding protein G(q) subunit alpha	GNAQ	YYLNDLDR	10.4	1.2	11.7	2.1
P19086	Guanine nucleotide-binding protein G(z) subunit alpha	GNAZ	IAAADYIPTVEDILR	11.3	1.1	10.2	2.1
P07359	Platelet glycoprotein Ib alpha chain	GP1BA	GQDLLSTVSIR	49.1	5.2	10.6	2.1
P07359	Platelet glycoprotein Ib alpha chain	GP1BA	LTSLPLGALR	45.2	2.9	6.4	2.1
P13224	Platelet glycoprotein Ib beta chain	GP1BB	LSLTDPLVAER	40.7	4.7	11.7	2.1
P40197	Platelet glycoprotein V	GPV	ITHLPGALLDK	13.1	1.1	8.4	2.1
P62993	Growth factor receptor-bound protein 2	GRB2	FNSLNELVDYHR	12.2	1.3	10.5	2.1
P05556	Integrin beta-1	ITB1	GEVFNELVGK	7.1	0.8	11.4	2.1
P05556	Integrin beta-1	ITB1	IGFGSFVEK	16.3	1.7	10.3	2.1
P05106	Integrin beta-3	ITB3	HVLTLTDQVTR	126.3	12.7	10.1	2.1
Q13976	cGMP-dependent protein kinase 1	KGP1	DLKPENLILDHR	2.2	0.2	9.0	2.1
Q05655	Protein kinase C delta type	KPCD	DYSNFDQEFLNEK	3.7	0.3	8.8	2.1
P43405	Tyrosine-protein kinase SYK	KSYK	KPFNRPQGVQPK	2.7	0.3	9.7	2.1
P43405	Tyrosine-protein kinase SYK	KSYK	NVLLVTQHYAK	2.9	0.2	8.3	2.1
Q13094	Lymphocyte cytosolic protein 2	LCP2	IQKPPLPPTTER	3.2	0.3	10.7	2.1
P18054	Arachidonate 12-lipoxygenase, 12S-type	LOX12	GEEEEFDHDVAEDLGLLQFVR	84.4	14.3	16.9	8.3
Q5SQ64	Lymphocyte antigen 6 complex locus protein G6f	LY66F	VYDVLVLK	13.5	0.9	6.7	2.1
P28482	Mitogen-activated protein kinase 1	MK01	GQVFDVGPR	3.1	0.3	8.3	0.5
Q15746	Myosin light chain kinase, smooth muscle	MYLK	VSDFYDIEER	6.0	0.4	7.0	2.1
O14745	Na(+)/H(+) exchange regulatory cofactor NHE-RF1	NHRF1	LLVVDPETDEQLQK	4.8	0.3	7.0	2.1
O14745	Na(+)/H(+) exchange regulatory cofactor NHE-RF1	NHRF1	SVDPDSPAEASGLR	5.2	0.4	7.8	2.1
Q9UFN0	Protein NipSnap homolog 3A	NPS3A	LVGVFHTEYGALNR	3.4	0.3	9.7	2.1
P47712	Cytosolic phospholipase A2	PA24A	NVSHNPLLLLTPQK	5.0	0.5	9.3	2.1
O76074	cGMP-specific 3′,5′-cyclic phosphodiesterase	PDE5A	GIVGHVAALGEPLNIK	10.0	1.0	9.7	2.1
P62136	Serine/threonine-protein phosphatase PP1-alpha catalytic subunit	PP1A	LNLDSIIGR	4.3	0.4	9.6	2.1
P62136	Serine/threonine-protein phosphatase PP1-alpha catalytic subunit	PP1A	NVQLTENEIR	3.0	0.2	7.9	2.1
P29350	Tyrosine-protein phosphatase non-receptor type 6	PTN6	IQNSGDFYDLYGGEK	6.2	0.4	6.4	2.1
Q12913	Receptor-type tyrosine-protein phosphatase eta	PTPRJ	VITEPIPVSDLR	3.0	0.2	7.4	0.5
Q07960	Rho GTPase-activating protein 1	RHG01	NPEQEPIPIVLR	3.3	0.2	6.9	2.1
O43182	Rho GTPase-activating protein 6	RHG06	SVPIQSLSELER	2.7	0.2	7.9	2.1
P12931	Proto-oncogene tyrosine-protein kinase Src	SRC	GPSAAFAPAAAEPK	2.3	0.2	8.5	2.1
Q9Y490	Talin-1	TLN1	ALDGAFTEENR	112.2	7.9	7.0	2.1
Q86YW5	Trem-like transcript 1 protein	TRML1	VSLNILPPEEEEETHK	49.7	6.7	13.5	2.1
**Platelet secretion**							
O00203	AP-3 complex subunit beta-1	AP3B1	VVNVANVGAVPSGQDNIHR	0.9	0.1	6.6	0.5
P54920	Alpha-soluble NSF attachment protein	SNAA	NSQSFFSGLFGGSSK	11.3	0.8	7.4	2.1
P54920	Alpha-soluble NSF attachment protein	SNAA	YEELFPAFSDSR	5.0	0.3	5.7	2.1
O95721	Synaptosomal-associated protein 29	SNP29	SVFGGLVNYFK	2.9	0.2	7.2	2.1
Q13586	Stromal interaction molecule 1	STIM1	SHSPSSPDPDTPSPVGDSR	1.5	0.2	14.4	0.1
Q13586	Stromal interaction molecule 1	STIM1	YAEEELEQVR	2.6	0.3	10.1	2.1
Q12846	Syntaxin-4	STX4	VALVVHPGTAR	0.7	0.1	16.6	0.5
O15400	Syntaxin-7	STX7	LVAEFTTSLTNFQK	21.4	2.8	13.3	2.1
O75558	Syntaxin-11	STX11	AQYNALTLTFQR	7.2	0.6	8.7	2.1
O75558	Syntaxin-11	STX11	LAELLDLSK	18.6	1.8	9.8	2.1
Q9P253	Vacuolar protein sorting-associated protein 18 homolog	VPS18	IEDVLPFFPDFVTIDHFK	2.9	0.2	7.8	2.1
**Platelet aggregation**							
P17301	Integrin alpha-2	ITA2	AIASIPTER	2.7	0.2	8.6	2.1
P17301	Integrin alpha-2	ITA2	ILGSDGAFR	3.2	0.3	9.8	2.1
P17301	Integrin alpha-2	ITA2	TQVGLIQYANNPR	3.2	0.3	8.6	2.1
P08514	Integrin alpha-IIb	ITA2B	IVLLDVPVR	170.6	10.2	6.0	2.1
Q9NS28	Regulator of G-protein signaling 18	RGS18	DGLEAFTR	2.6	0.2	6.9	2.1

## Supplementary Materials

The following are available online www.mdpi.com/2227-7382/5/4/31/s1, Table S1: Results of the least squares linear regression, Table S2: Endogenous concentration of all measured platelet peptides, Table S3: Raw results and residuals of the concentration curves, Table S4: Time and percental concentration of solvent B in the optimized gradient.
